# Improvement on Ferrous Ion Accumulation and Mitochondrial Dysfunction in the COVID-19 Pseudovirus-Infected Cell Model Simulating the Long COVID Status by Nutritional Strategy

**DOI:** 10.3390/life15060980

**Published:** 2025-06-18

**Authors:** Bo-Kai Chen, Chi-Ho Chan, Chin-Kun Wang

**Affiliations:** 1 Department of Nutrition, Chung Shan Medical University, Taichung 402, Taiwan; gargon88@gmail.com; 2 Department of Microbiology and Immunology, Chung Shan Medical University, Taichung 402, Taiwan; 3 Department of Medical Research, Chung Shan Medical University Hospital, Taichung 402, Taiwan

**Keywords:** long COVID, pseudovirus, ferrous ion, mitochondrial dysfunction, lactoferrin, Q10, *Echinacea purpurea* extract

## Abstract

The pandemic caused by the severe acute respiratory syndrome coronavirus 2 (SARS-CoV-2) has plunged the world into a major crisis of overwhelming morbidity and mortality and emerged various mutant strains. Patients recovering from SARS-CoV-2 develop post-acute COVID syndrome, commonly known as long COVID (LC), lasting up to 12 weeks or even longer. The mechanism has yet to be clarified. COVID-19 pseudovirus is a suitable model to understand the infection of the COVID-19 virus to cells, which is suitable to see the acute change in cells owing to its one-time infection and inactivation. The ACE2-293T cell infected by COVID-19 pseudovirus was used in this study. After the infection and removal of the pseudovirus, high amounts of ferrous ions were accumulated in mitochondria and then released into the cytosol. Reactive oxygen species (ROS) accumulation was formed and caused mitochondrial dysfunction. To evaluate the effect of nutritional strategy on ferrous ion accumulation and mitochondrial dysfunction, lactoferrin, Q10 and *Echinacea purpurea* extract (EPE) were used in this study. Results showed that lactoferrin, Q10 and EPE could improve mitochondrial dysfunction by reducing the accumulation of ferrous ions and ROS in the mitochondria. HPLC analysis showed that EPE contained rich caffeic acid, and it also showed perfect improvement in mitochondrial dysfunction. In conclusion, cells infected with pseudovirus could increase the accumulation of ferrous ions and ROS in mitochondria and be released into the cytosol after removing pseudovirus, thereby causing mitochondrial dysfunction. Lactoferrin, Q10 and EPE were an effective nutritional strategy to suppress ferrous ion accumulation, ROS formation and advanced mitochondrial dysfunction.

## 1. Introduction

### 1.1. Long COVID

Clinically, the severity of illness caused by SARS-CoV-2 can range from asymptomatic to fatal [1]. A subset of patients infected with SARS-CoV-2 present with persistent distressing symptoms and/or new symptoms after infection. It is broadly classified into post-COVID syndrome, post-acute sequelae of SARS-CoV-2 (PASC) or long COVID (LC) [2]. LC symptoms include severe and recurring fatigue, difficulty breathing, chest tightness, cough, brain fog and headache [3]. According to statistics, approximately 25–70% of virus-free COVID-19 survivors continued to experience human metabolic reprogramming/dysregulation (HMRD) caused by the COVID-19 infection. These symptoms were persistent, exacerbated or new clinical events, collectively referred to as post-acute sequelae of COVID-19 (PASC) or LC [4]. People with LC experience multiple symptoms simultaneously, with some cases reporting more than 200 different and overlapping symptoms that may last for weeks, months or even years. However, the pathogenesis of this heterogeneous disease is still unclear, and there are no diagnostic criteria and definitions for LC. Therefore, it is very important to establish diagnostic biomarkers and strategies [5].

### 1.2. The Importance of Mitochondria in Viral Infections

Clinically, it has been found that many patients with COVID-19 experience a surge in inflammatory events, some even with fatal consequences. A high level of inflammation is associated with oxidative stress, dysregulated iron homeostasis, hypercoagulability and thrombosis [6,7]. Oxidative stress is considered a major factor in the pathogenesis and severity of COVID-19 [8]. Hyperferremia has also been highlighted as a predictor of increased mortality from this disease [9]. Previous studies emphasized the role and management of iron status dysregulation in the pathogenesis of COVID-19 [4,10].

Mitochondria is a vital organelle in cells. Its function involves cellular respiration, energy production, regulation of cellular metabolic processes and production of reactive oxygen species (ROS) [11]. Mitochondria have been implicated in antiviral immunity, which could modulate inflammatory response [12]. Studies have shown that many viral infections can directly or indirectly affect mitochondrial function, which involves cellular energy metabolism and biosynthesis pathways, thus affecting antiviral immune responses. Therefore, mitochondria play a very important role in resisting viral infection [13]. Therefore, normal mitochondrial function can enhance cell viability against viral infection [14]. The vicious cycle of abnormal states such as inflammatory cytokine storm, oxidative stress, microbiota dysbiosis, iron overload and ROS accumulation could lead to intra- and extra-mitochondrial dysfunction [15]. Continuous mitochondrial dysfunction could induce cell reprogramming and cause different kinds of syndromes and discomfort. This shows that LC has an association with mitochondrial dysfunction. Nutritional strategy could be an effective choice for improving mitochondrial dysfunction.

### 1.3. Nutritional Strategy

Lactoferrin is a member of the transferrin family, a 70 kDa glycoprotein. It has a very high affinity with two iron ions (Fe^3+^), converting the open apo-LF configuration into the closed holo-LF configuration [16]. Iron can be absorbed by the body in two forms, heme (from meat) and non-heme, which is essential for cytochromes, peroxidases, nitric oxide synthase and other important functions involved in biosynthesis and energy production, detoxification, host defense, cell signaling, etc. [17]. Non-heme iron is absorbed through nonspecific transport with the participation of divalent metal ion transporter-1, mainly in the form of reduced Fe^2+^, and eventually enters the body in the form of oxidized Fe^3+^ [18]. However, the bioavailability of Fe^3+^ must rely on binding with proteins or other hydrophilic chelators [17].

Q10 or ubiquinone (2,3-dimethoxy-5-methyl-6-polyisoprene-1,4-benzoquinone) is a lipophilic molecule that plays many key roles in the human body. For example, the antioxidant effect of Q10 is closely related to cell function and cell viability [19]. Studies have also pointed out that Q10 plays a key role in regulating ferroptosis [20]. Q10 can increase mitochondrial mass, improve mitochondrial function, and inhibit ROS generation in the mitochondrial electron transport chain [21].

*Echinacea purpurea* (EP), commonly known as purple coneflower, is native to North America and has been used to treat a variety of infectious diseases and wounds [22]. EP is a medicinal plant with potent immunostimulatory properties [23]. The most well-known phytochemicals in EPs are phenolic compounds, such as caffeic acid derivatives: caffeic acid, chlorogenic acid, cynarin, echinacea glycosides and chicoric acid [24]. Our previous study showed that EPE can prevent viral entry into cells by improving cell viability and mitochondrial dysfunction and exhibiting direct inhibitory effects on viral infection [25]. Therefore, this study continued to conduct a pseudovirus infection study on EPE.

This study used a cell model infected with COVID-19 pseudovirus and then removed the pseudovirus, and used the characteristics of one-time infection and inactivation to simulate the changes in mitochondria after real COVID-19 infection. Then, it analyzed the phenolic components in EPE by HPLC and determined the improvement of mitochondrial dysfunction by using lactoferrin, Q10, EPE and the phenolic component in EPE to confirm the contributor.

## 2. Materials and Methods

### 2.1. Cell Lines and Pseudovirus

The culture method of angiotensin I-converting enzyme 2 293T cells (ACE2-293T) was referenced from [26]. The cells were cultured at 37 °C and 5% CO_2_ using Dulbecco’s modified Eagle’s medium (DMEM, Gibco, Billings, MT, USA, containing 10% fetal bovine serum and 1% penicillin/streptomycin). ACE2-293T cell line was synthesized from a human embryonic kidney-derived cell line (293T cell line) that constitutively expresses human angiotensin I-converting enzyme 2 (ACE2) under the cytomegalovirus (CMV) promoter (Takara Bio USA, Inc., San Jose, CA, USA, NO. 631289).

The pseudovirus used in this study is a synthetic virus with the same structure as the COVID-19 virus and infects cells by binding to the ACE2 receptor. The synthetic materials of pseudovirus include SARS-CoV-2 (Wuhan strain) spike plasmid, HIV-Core plasmid and HEK-293T cell line (American Type Culture Collection, Manassas, VA, USA, No.CRL-11268). The structure of a pseudovirus was composed of the core of an enveloped virus and the envelope glycoprotein of another enveloped virus. The spike is responsible for recognizing the cell surface receptor ACE2 and mediating the entry of the virus. The envelope glycoprotein of SARS-CoV-2 is the spike protein on the surface of the pseudovirus in this study. The pseudovirus synthesis method used in this study was based on the description of [27]. Pseudoviruses carrying the S glycoprotein and the firefly luciferase (FLuc) reporter gene were generated in HEK-293T cells. 5 μg of pCMVΔR8.2, 5 μg of pHR’CMVLuc and 0.5 μg of S or its mutants (codon-optimized) expression plasmids were cotransfected into cells with or without 2 μg of TMPRSS2 expression plasmid. After approximately 48 h of incubation, pseudovirus supernatants were collected and filtered through a 0.45 μm low protein binding filter and stored at −80 °C as described previously [28].

### 2.2. Timeline of Experimental Design

**Figure 1:**
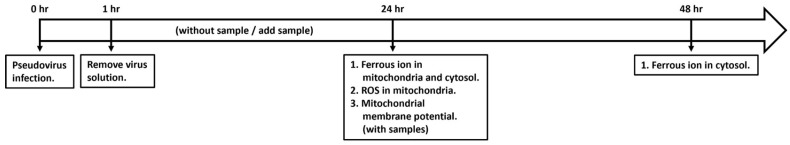
Timeline of experimental design for cell exposure to pseudovirus and intervention of antioxidant nutrients.

Figure 1 shows the timeline of the experimental design. The cells were infected with pseudovirus for 1 h, and then the pseudovirus solution was removed to ensure that no pseudovirus solution remained in the culture medium. The accumulation of ferrous ions and formed ROS in mitochondria and the mitochondrial membrane potential were observed 24 h after culture. The accumulation of ferrous ions in the cytosol was observed 24 and 48 h after culture.

### 2.3. Testing Samples

The lactoferrin and Q10 used in this study were provided by Bioquad Life Sciences Co., Ltd., Yorba Linda, CA, USA, dissolved in a cell culture medium to form a stock solution and stored at 4 °C. The stock solution was diluted for use in subsequent experiments. EP was extracted with hot water to obtain EPE and provided by Standard Foods Co., Ltd., Taipei, Taiwan. After solid–liquid phase separation, the supernatant was taken, filtered and packaged.

### 2.4. HPLC Analysis of Phenolic Compounds

The chromatographic conditions for the analysis of phenolic compounds in EPE were set according to the parameters in [29]. The analytical columns were SuperSpher1 100, RP-18 cartridge (75 × 4.6 mm i.d.; 3 mm; BDH, Toronto, ON, Canada) and SuperSpher 100, RP-18 guard cartridge (4 × 4.6 mm i.d.; 5 mm). The mobile phase consisted of 50 mM sodium dihydrogen phosphate (solvent A) adjusted to pH 2.8 with phosphoric acid and 1% 0.1 M phosphoric acid in acetonitrile (solvent B). The elution curve was as follows: a linear gradient from 5% to 25% solvent B in 7 min, maintained at 25% for 2 min, then decreased from 25% to 5% solvent B in 1 min, and finally equilibrated with 5% solvent B for 5 min, with a flow rate of 1.5 mL/min and a detection wavelength of 320 nm.

### 2.5. Cytotoxicity Test

The cytotoxicity assay method in this study was based on the previously described sulforhodamine B (SRB) assay with some modifications [30,31]. The cells were cultured in a medium containing 10% fetal bovine serum and seeded in 96-well plates. The testing samples were prepared with serially two-fold dilution from a stock solution. For the control group, 100 μL of growth medium was added to each corresponding well for sextuplicate. For the treatment groups, 100 μL of samples of different concentrations were added to each corresponding well for sextuplicate. The cells were co-cultured with samples at 37 °C, 5% CO_2_ for 24 h. Then 10 μL of Cell Counting Kit-8 (CCK-8) (Dojindo, Mashiki, Japan) was added to each well. The well was mixed to obtain a homogenous solution by gently tapping the outside of the plate several times to avoid creating bubbles. The cells were incubated with the CCK-8 kit at 37 °C and 5% CO_2_ for 1 h until the color change. The absorbance at 450 nm was measured by using an enzyme-linked immunosorbent analyzer (ELISA reader) (Agilent BioTek Synergy H1, Santa Clara, CA, USA), and the results were interpreted as a reference wavelength of 650 nm [32]. The average value of the control group was used as the reference value, and the average absorbance values of each group were converted into percentages for comparison.

### 2.6. Staining of Ferrous Ions in Mitochondria and Cytosol by Ferro Orange/Green

To detect intracellular and mitochondrial Fe^2+^, staining was performed using FerroOrange and FerroGreen (Dojindo) as described previously [33]. After staining, cells were washed twice with Hank’s balanced salt solution (HBSS) to remove residues. Cells were then treated with 1 μM FerroOrange or 5 μM FerroGreen for 30 min at 37 °C. After staining, the cells were washed three times with HBSS to remove the stain and fluorescent images were obtained by fluorescent microscope.

### 2.7. ROS Production in Mitochondria by MitoSOX Red Staining

The method used in this study was modified from that of modification [34]. Cells were co-labeled with 100 nM photosensitizer and a live cell ROS fluorescent indicator (5 μM MitoSOX Red [Invitrogen, Carlsbad, CA, USA, M36008]) in cell culture medium at 37 °C for 30 min. After the incubation, cells were washed with HBSS buffer to remove the staining solution. Fluorescent images were collected by fluorescent microscope.

### 2.8. Analysis of Mitochondrial Membrane Potential by JC-1 Staining

The protocol and method referred to the previous study of [35]. The cells were cultured in a medium containing 10% fetal bovine serum and prepared to confluence in 24-well plates. The cells were incubated with assay buffer containing JC-1 at 37 °C for 30 min. At the end of the incubation period, cells were washed to remove the staining solution, and fluorescent images were collected using an inverted fluorescence microscope. The results were measured for red and green fluorescence in each group by using Image J software (https://imagej.net/ij/download.html, accessed on 16 June 2025; National Institute of Mental Health, Bethesda, MD, USA) and expressed as the ratio of red/green fluorescence intensity.

### 2.9. Statistical Analysis

Statistical analysis was performed using the SPSS 25.0 software package. A Shapiro-Wilk test was performed to examine the normality of the results. All statistical data are shown as the mean ± standard error (SEM). Statistical significance was verified using an ANOVA, followed by a post hoc Tukey’s test. *p* < 0.05 indicated statistical significance.

## 3. Results

### 3.1. Effects of Pseudovirus Infection on Ferrous Ions Accumulation in Mitochondria and Release into Cytosol

**Figure 2:**
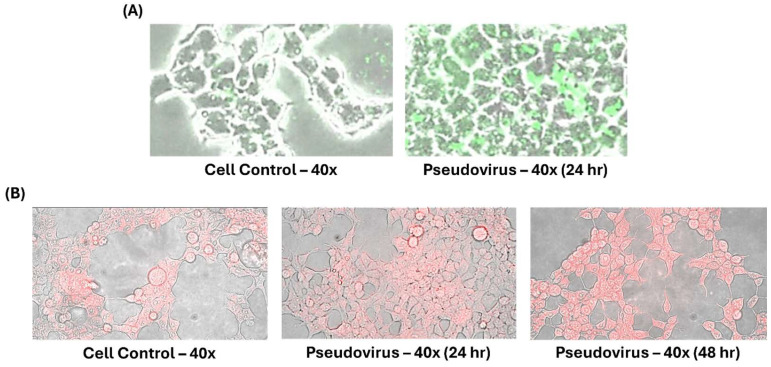
Ferrous ion accumulation after pseudovirus infection. Different stains were used to stain ferrous ions in mitochondria and cytosol. (**A**) Green staining represents ferrous ions in the mitochondria. (**B**) Red staining represents ferrous ions in the cytosol.

Cells were infected with pseudovirus for 1 h after removing the pseudovirus solution. Infected cells were washed with PBS solution to remove the remaining pseudovirus solution and continuously cultured with culture medium for 24 h at 37 °C and 5% CO_2_. We observed the accumulation of ferrous ions in the mitochondria and cytosol at different time points to confirm the change in mitochondria and cytosol after pseudovirus infection. FerroGreen staining showed the increase of ferrous ions level in mitochondria after 24 h of incubation (Figure 2A). Figure 2B shows the accumulation of ferrous ions in the cytosol after 24 and 48 h of incubation. FerroOrange staining clearly induced more accumulation of ferrous ions at 48 h than those at 24 h. It is speculated that accumulated ferrous ions in mitochondria could be released after longer incubation in the cytosol.

### 3.2. The Effect of Change in Mitochondrial Oxidative Status on Mitochondrial Membrane Potential

**Figure 3:**
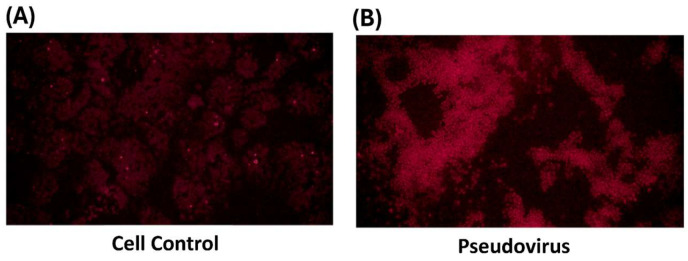
Superoxide accumulation in mitochondria by MitoSOX red staining. The red staining represents the level of ROS in mitochondria. (**A**) in control cells, (**B**) in infected cells.

**Figure 4:**
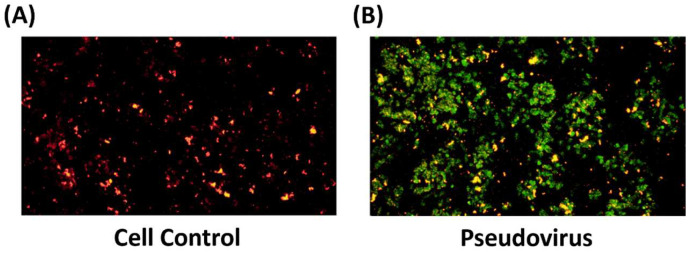
Double fluorescence staining of mitochondrial membrane potential by JC-1. (**A**) in control cells, (**B**) in infected cells. Green fluorescence indicates decreased membrane potential, representing mitochondrial dysfunction. Red fluorescence indicates the increase in mitochondrial membrane potential. The results showed that the mitochondrial membrane potential changed after cells were infected with pseudovirus.

To confirm the change in oxidative status within mitochondria after infection. The level of ROS in mitochondria after 24 h of pseudovirus infection was observed and showed an increase of ROS in mitochondria (Figure 3). After measuring ferrous ion and ROS accumulation in mitochondria, mitochondrial membrane potential was determined to confirm the impact of pseudovirus infection. Results showed a strong increase in green fluorescence after infection by JC-1 staining (Figure 4).

### 3.3. Cytotoxicity Test

Ferrous ion accumulation and the increase of ROS in mitochondria could be resolved by antioxidant nutrients. Lactoferrin, Q10 and EPE were used as the nutritional strategy in this study. After the cells were treated with different samples and cultured with CCK-8, the absorbance values were obtained, and the average value of the control group was used as a reference to obtain the cytotoxicity percentage of each group. Noncytotoxic concentrations were defined as more than 80% of surviving concentrations. The highest noncytotoxic concentrations were 50 mg/mL for lactoferrin, 20 mg/mL for Q10 and 2 mg/mL for EPE. Further studies were performed under the noncytotoxic concentrations.

### 3.4. Improvement in Ferrous Ion Accumulation

**Figure 5:**
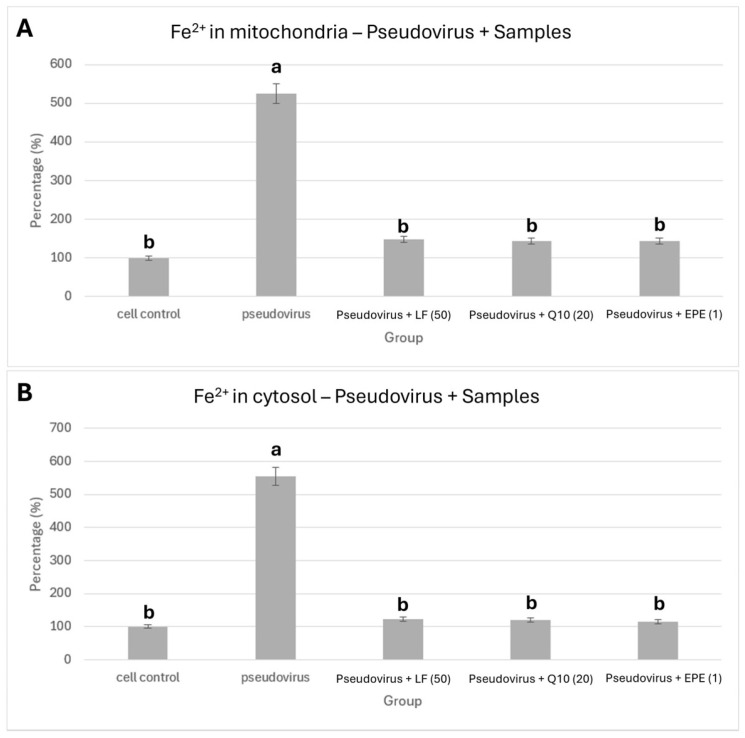
Effect of lactoferrin, Q10 and EPE on ferrous ion accumulation. (**A**) in mitochondria, (**B**) in cytosol. Cells were treated with pseudovirus for 1 h. The pseudovirus solution was discarded, then washed with PBS, and testing sample solution was added and cultured for 24 h. The testing samples are as follows: LF (50), Lactoferrin 50 mg/mL; Q10 (20), 20 mg/mL; and EPE(1), *Echinacea purpurea* extract, 1 mg/mL. Data is shown as mean % relative to cell control ± SEM. Different letters denote significant differences (*p* < 0.05, one-way ANOVA with post hoc Tukey’s test).

Figure 5A and Figure 5B, respectively, show the improvement in ferrous ions in mitochondria and cytosol by treating with 50 mg/mL lactoferrin, 20 mg/mL Q10 and 2 mg/mL EPE. Figure 5A shows that antioxidant nutrients, lactoferrin (525.1% vs. 147.9%), Coenzyme Q10 (525.1% vs. 144.1%) and EPE (525.1% vs. 144.4%) significantly reduced the accumulation of ferrous ions in mitochondria compared with pseudovirus group (*p* value < 0.01). Figure 5B also shows that lactoferrin (554.4% vs. 123.0%), Coenzyme Q10 (554.4% vs. 120.7%) and EPE (554.4% vs. 114.7%) could significantly improve the accumulation of ferrous ions in the cytosol (*p* value < 0.01).

### 3.5. Improvement of Mitochondrial Dysfunction

**Figure 6:**
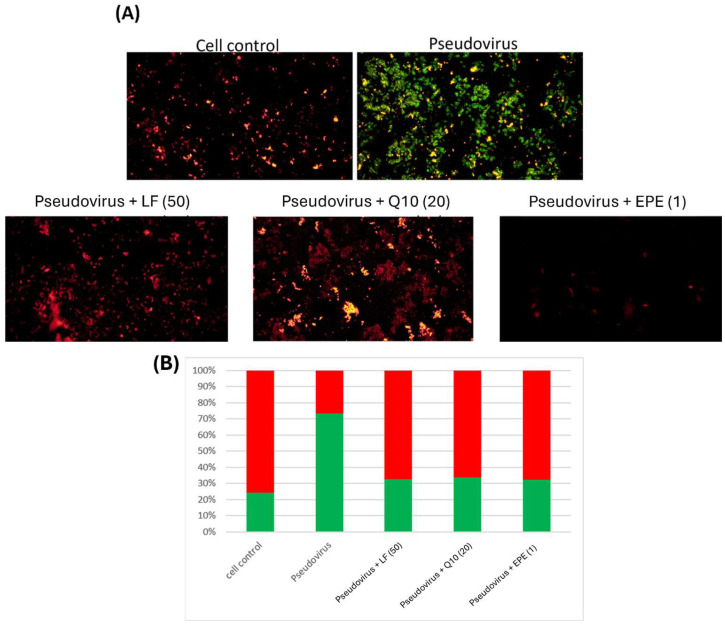
Double fluorescence staining of mitochondrial membrane potential by JC-1 after the intervention of lactoferrin, Q10 and EPE. (**A**) Mitochondrial membrane potential morphology in cells. (**B**) Proportion of red and green fluorescence. The results showed an improvement in mitochondrial dysfunction after intervention with different samples. Green fluorescence indicated the decreased membrane potential, and red fluorescence indicated that samples effectively preserve the mitochondrial membrane potential. The following testing samples are as follows: LF (50), Lactoferrin 50 mg/mL; Q10 (20), 20 mg/mL; and EPE (1), *Echinacea purpurea* extract, 1 mg/mL.

Lactoferrin, Q10 and EPE were also used to evaluate the improvement in mitochondrial dysfunction. Figure 6A shows the mitochondrial morphology of infected cells treated with different samples. Figure 6B indicates that lactoferrin, Q10 and EPE significantly repaired the damage of mitochondrial membrane potential by pseudovirus infection. This clearly shows that nutritional strategy could effectively improve mitochondrial dysfunction induced by pseudovirus infection.

### 3.6. Phenolic Compounds in EPE

**Figure 7:**
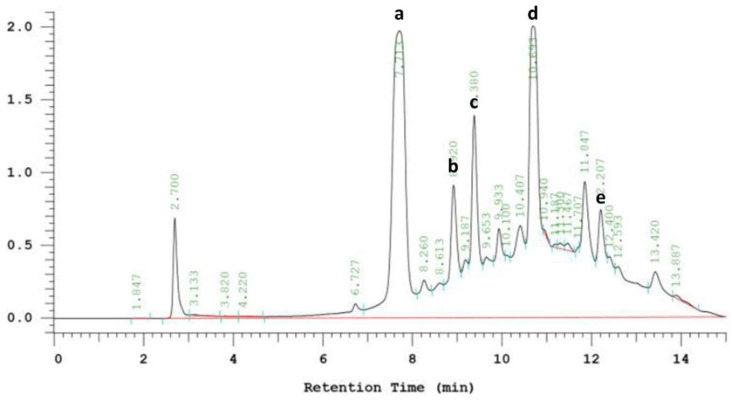
HPLC chromatogram of phenolic compounds in *Echinacea purpurea* extract (EPE). Detected at 320 nm. (**a**) Chlorogenic acid. (**b**) Echinacoside. (**c**) Cichoric acid. (**d**) Caffeic acid. (**e**) Cynarin.

EPE is a natural phytochemical extract. Figure 7 shows the chromatogram of phenolic compounds in EPE. The EPE contained chlorogenic acid, echinacoside, cichoric acid, caffeic acid and cynarin. Caffeic acid was the richest one (Table 1). Ref. [36] found that caffeic acid derivatives exhibited bioactivities.

The main caffeic acid derivatives in EP are caffeic acid, chlorogenic acid, caffeic acid, cynarin, echinacoside and chicoric acid. Among them, chicoric acid and echinacoside have the most significant biological activities and are considered to be the active phytochemicals in EP.

### 3.7. Effect of Caffeic Acid on Mitochondrial Dysfunction

**Figure 8:**
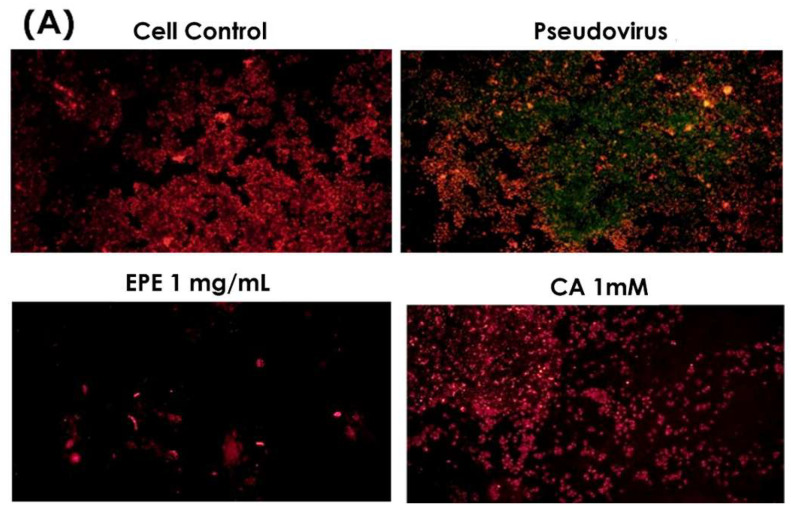

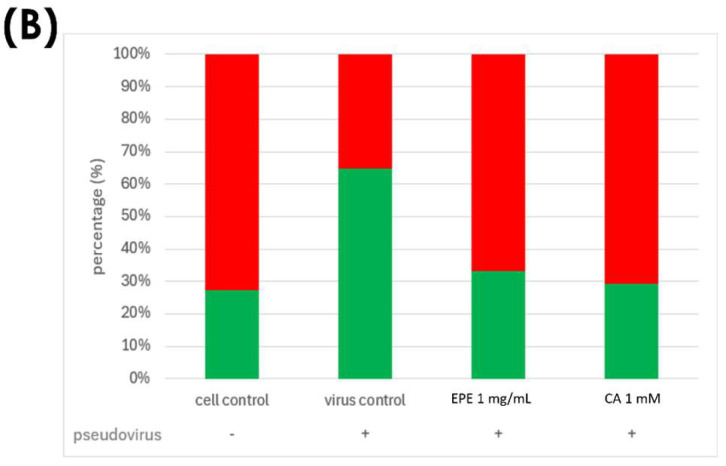
Double fluorescence staining of mitochondrial membrane potential by JC-1 after treatment with CA1000 and EPE (1). (**A**) Mitochondrial membrane potential morphology in cells. (**B**) Proportion of red and green fluorescence. Green fluorescence indicated the decreased membrane potential, and red fluorescence indicated that samples effectively preserve the mitochondrial membrane potential. EPE 1 mg/mL, *Echinacea purpurea* extract 1 mg/mL; CA 1 mM, Caffeic acid 1 mM.

Based on the quantification of phenolic compounds in EPE, an equivalent concentration of caffeic acid 1.54 mM in 1 mg/mL of EPE was used to evaluate its effect on mitochondrial membrane potential change. Figure 8 clearly shows that caffeic acid greatly reduced green fluorescence, similar to that of 1 mg/mL of EPE. Therefore, it is speculated that caffeic acid is one of the contributors to EPE.

## 4. Discussion

Respiratory viral infections are a group of diseases that affect millions of people worldwide, with children and the elderly being particularly vulnerable. In general, respiratory viral infections are mostly associated with cytokine production, inflammation, cell death and other pathophysiological processes that may be related to redox imbalance or oxidative stress (OS). It is known that excessive production of ROS and loss of antioxidant mechanisms are inextricably linked to viral replication and subsequent virus-related diseases [37]. OS is caused by the disruption of the prooxidant-antioxidant balance, which results in the dominance of prooxidants and leads to cell damage [38]. High levels of ROS and OS are associated with pollutants, toxins and viral infections in the respiratory tract, ultimately leading to cellular damage. Some respiratory viruses have been shown to cause increased recruitment of inflammatory cells to the site of infection, leading to dysregulated ROS formation. In addition, ref. [39] also pointed out that viral infection can lead to an imbalance in the oxidative-antioxidant status, thereby causing oxidative cell damage. It is noteworthy that the levels of cytokines and chemokines in the blood of patients infected with COVID-19 were significantly increased, and the cytokine storm triggered a pro-inflammatory environment, which was closely related to severe tissue damage and led to the death of COVID-19 patients [40]. Clinically, it has also been found that excessive production of ROS and deficiency of the antioxidant system play an important role in the pathogenesis of SARS-CoV infection and the progression and severity of respiratory diseases. An experimental animal model of severe acute respiratory syndrome also found that ROS levels were increased and antioxidant defenses were impaired during SARS-CoV infection [41]. Some of the literature also indicated that SARS-CoV protease (protein 3a) has also been implicated in the activation of the mitochondrial cell death pathway [42].

Clinically, interferon (IFN) treatment has been found to reduce the duration and severity of symptoms, as well as reduce mortality [43]. The use of IFN-γ for viral infections such as hepatitis C is associated with symptoms such as fever, diarrhea, headache, chills, nausea, myalgia and/or fatigue [44]. These symptoms are very similar to those of LC. Moreover, some studies have pointed out that inefficient or dysfunctional immune response is related to chronic fatigue after infection [45]. Ref. [15] also found a correlation between iron dysregulation and disease severity. Iron dysregulation induced ROS production and promoted oxidative stress. Elevated inflammation/oxidative status may lead to mitochondrial dysfunction, leading to platelet damage and apoptosis. This study showed similar results in a pseudovirus-infected cell model and caused the accumulation of ferrous ions in mitochondria and speculated that accumulated ferrous ions in mitochondria could be released after a longer time into the cytosol, also causing an increase in ROS in mitochondria. These changes could increase the risk of mitochondrial dysfunction and affect cellular function. Infected cells in this study also showed a significant loss of mitochondrial membrane potential.

LC, or PASC, is an important long-term clinical problem caused by COVID-19 infection. A large number of people infected with COVID-19 places a huge burden on health services. Therefore, it is crucial to understand the pathogenic mechanisms behind LC. In this study, we used a pseudovirus-infected cell model for discussion. Results showed that after pseudovirus infection, it could greatly increase the accumulation of ferrous ions and ROS in mitochondria and cause mitochondrial membrane potential change. A slight increase in inflammatory cytokine levels (TNF-α, IL-6 and IL-1β) was also found. These were similar to previous studies. LC patients showed slightly increased secretion of IL-1β, IL-6, TNF-α, GM-CSF and G-CSF, raising the possibility that other pro-inflammatory cytokines may also be disturbed, as observed by others 8 months after infection [46].

Excessive inflammation, elevated pro-inflammatory cytokines and stimulation of ferritin and hepcidin synthesis (ultimately mediating FeRD) are hallmarks of severe COVID-19 infection [10]. Disturbance in iron homeostasis is manifested by high iron levels in the reticuloendothelial cells and increased serum ferritin concentrations. When the iron-binding capacity of transferrin (TF) in the blood exceeds normal levels, free iron is released into the plasma in a redox-active state, called labile plasma iron (LPI), forming free radicals that damage tissues and lead to fibrosis [47]. Clinically, it has been found that a ferritin/TF ratio > 10 is associated with a 5-fold increased risk of ICU admission and an 8-fold increased risk of mechanical ventilation in COVID-19 patients [48]. Ref. [49] also pointed out that biomarkers of iron metabolism (i.e., ferritin, transferrin (TF), lactoferrin (LF), etc.) and hemoglobin may be used as indicators to provide risk stratification strategies for the management of COVID-19. Compared with the results of this study, abnormal mitochondrial iron metabolism may be a strong indicator of persistent symptoms after COVID-19 infection.

Studies have shown the antiviral activity of chicoric acid [50] and has been found to inhibit HIV-1 integrase and replication [51]. Among caffeic acid derivatives, although echinacoside does not have immunostimulatory activity, it has the potential for antioxidant activity and protects collagen from the effects of reactive oxygen species [52,53]. Caffeic acid (3,4-dihydroxycinnamic acid) is a plant-abundant polyphenolic compound containing two phenolic hydroxyl groups and is the major metabolite produced by the hydrolysis of chlorogenic acid. Previous studies have shown that caffeic acid has an inhibitory effect on influenza A virus proliferation, which may be due to specific interactions of caffeic acid with cellular and/or viral proteins involved in viral genome replication [54]. Cynarin has also shown several pharmacological properties, including cholesterol-lowering [55], hepatoprotective [56], antiviral, antibacterial and antihistamine [57] activities.

According to the results of this study, LC could begin from the accumulation of ferrous ions and ROS in mitochondria and induce mitochondrial dysfunction. This also suggests that cell reprogramming could cause different problems. To treat this problem, nutritional strategy could be effective instead of medical intervention. In this study, lactoferrin, Q10 and EPE were used to treat pseudovirus-infected cells and showed the improvement of mitochondrial dysfunction. Since caffeic acid is the most abundant component of EPE, results also showed that caffeic acid had an effective improvement on mitochondrial dysfunction and was similar to EPE.

Previous studies showed that a high frequency of IFN-γ release persisted in patients with LC even beyond 180 days of symptom onset [58]. Clinically, it has been found that the use of IFN-γ to treat viral infections such as hepatitis C can cause symptoms such as fever, diarrhea, headache, chills, nausea, muscle pain and/or fatigue. These symptoms are similar to those of patients with LC, and it is speculated that abnormal IFN-γ production may be a predictor for LC. For future work, it is worth studying whether patients with LC also showed high IFN-γ secretion and whether it can be used as an indicator to determine LC. If this is the case, then it could be a biomarker with broader clinical utility.

Despite the promising findings demonstrated in this study, several limitations should be acknowledged. First, the experimental model utilized a pseudovirus infection system in ACE2-293T cells to simulate SARS-CoV-2 infection and LC-like cellular responses. While this approach effectively mimics certain viral entry and post-infection events, it does not replicate the full spectrum of host immune interactions, viral replication dynamics or systemic physiological responses seen in vivo. Therefore, extrapolation of these findings to clinical settings must be carried out with caution. Second, the study relied exclusively on in vitro assays to evaluate the efficacy of nutritional interventions such as lactoferrin, Q10 and EPE. Although these results provide mechanistic insights, they lack the complexity of whole-organism responses, including metabolic variability, bioavailability and pharmacokinetics. Future animal model studies and clinical trials are necessary to validate the translational relevance of these findings. Third, while caffeic acid was identified as a major bioactive compound in EPE and showed improvement in mitochondrial dysfunction, the synergistic or antagonistic effects of other phenolic constituents were not fully characterized. The therapeutic contribution of the complete phytochemical profile remains to be elucidated. Furthermore, the concentrations of lactoferrin, Q10 and EPE used were relatively high; in the absence of in vivo data, their translational relevance remains uncertain. Lastly, this study focused primarily on mitochondrial dysfunction and oxidative stress parameters. While this study showed improvements in mitochondrial parameters with nutritional strategies, the pathways linking nutritional compounds to these effects have not been explored at the molecular level. Future studies will continue to explore the association between COVID-19 and ferroptosis regulation and Nrf2 pathway activation. Additional indicators of long COVID pathology, such as chronic inflammation, immune dysregulation and neurocognitive effects, were not assessed and represent important directions for future research.

## 5. Conclusions

In this study, it was hypothesized that mitochondria play a potentially important role after the infection of pseudoviruses. LC could begin from ferrous ion and ROS accumulation in mitochondria and induce mitochondrial dysfunction. This could be treated precisely by nutritional strategy. Based on the properties of ferrous ion accumulation, ROS and improved mitochondrial dysfunction, lactoferrin, Q10 and EPE showed positive effects. The in vitro results of this study show that nutritional strategy may be a new option for improving LC status. However, animal model studies and clinical trials are still needed to verify the translational application value of these findings. The pathways linking nutritional compounds to these effects have not yet been explored at the molecular level. In the future, we will continue to test other natural extracts with strong antioxidant properties and continue to explore the association between LC and ferroptosis regulation and Nrf2 pathway activation. Other long COVID pathological indicators, such as chronic inflammation, immune dysregulation and neurocognitive effects, are important directions for future research as indicators for improving LC.

## Figures and Tables

**Table 1:** Quantification of phenolic compounds in EPE.

	mg/g Dry Weight
Chlorogenic acid	131.15 ± 6.56
Echinacoside	37.03 ± 1.85
Cichoric acid	40.26 ± 2.01
Caffeic acid	261.48 ± 13.07
Cynarin	14.50 ± 0.72

EPE, *Echinacea purpurea* extract.

## Data Availability

The original contributions presented in this study are included in the article. Further inquiries can be directed to the corresponding author.
